# Mapping of electromagnetic waves generated by free-running self-oscillating devices

**DOI:** 10.1038/s41598-017-09802-0

**Published:** 2017-08-23

**Authors:** Shintaro Hisatake, Hikaru Nakajima, Hai Huy Nguyen Pham, Hirohisa Uchida, Makoto Tojyo, Yoichi Oikawa, Kunio Miyaji, Tadao Nagatsuma

**Affiliations:** 1 Gifu University, Department of Electrical, Electronic and Computer Engineering, Gifu, 501-1193 Japan; 2 Osaka University, Department of Systems Innovation, Osaka, 560-8531 Japan; 3 Arkray Inc., Kyoto, 602-0008 Japan; 4 Think-Lands Co., Ltd., Yokohama, 230-0046 Japan

## Abstract

Near-field mapping has proven to be a powerful technique for characterizing and diagnosing antennas in the microwave frequency range. However, conventional measurement methods based on a network analyzer cannot be applied to on-chip antenna devices extensively studied for future wireless communication in the millimeter wave (mm-wave) (30–300 GHz) and terahertz (THz) wave (0.1–10 THz) frequency regions. Here, we present a new asynchronous mapping technique to investigate the spatial distribution of not only the amplitude but also the phase of the electric field generated by free-running, self-oscillating generators including CMOS oscillators, Gunn oscillators, resonant tunneling diodes, and quantum cascaded lasers. Using a photonic-electronic hybrid measurement system, a wide frequency coverage, minimal invasiveness of the field to be measured, and phase distribution measurements with a theoretically-limited sensitivity are simultaneously achieved. As a proof-of-concept experiment, we demonstrate the mapping of a mm-wave (77 GHz) generated by a free-running Gunn oscillator and antenna characterization based on near-to-far field transformation.

## Introduction

Of late, millimeter (mm) and terahertz (THz) waves have attracted significant attention owing to their applications in radar systems^1^, 5 G networks^2^, THz wireless communication^3^, etc. In these higher frequency regions, on-chip antenna devices, in which the antennas are integrated with peripheral circuits such as oscillators, mixers, and amplifiers, have been extensively studied^4^. In addition to antenna-integrated electronics-based generators or devices, there has been a rapid development in photonics-based generators or devices^5^ intended to be integrated with antennas in the near future. A high output power is always required for the generators; additionally, directivity and beam quality become more important in these higher frequency regions compared to the microwave region, for achieving high-quality data transmission in wireless communication systems or for accurate target detection in radar applications.

**Figure 1:**
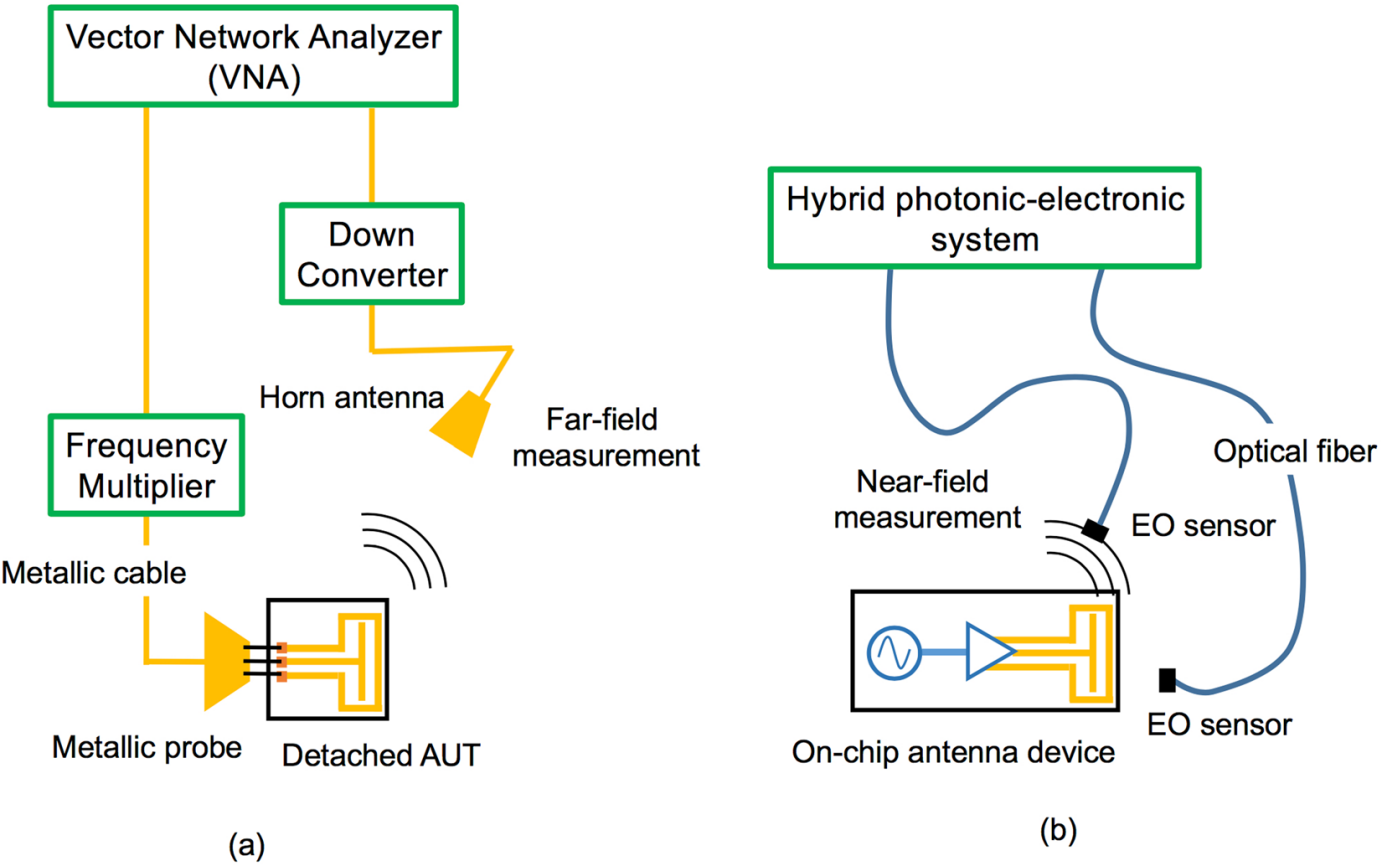
Comparison of the conventional and proposed measurement schemes. (**a**) Conventional system using a network analyzer and the (**b**) proposed system.

A measurement technique is essential for application development in any electromagnetic frequency region. Near-field mapping has proven to be a powerful technique for characterizing and diagnosing antennas in the microwave frequency range^6, 7^. In a conventional antenna measurement setup (Fig. 1(a)), the reference RF signal from a vector network analyzer (VNA) is fed to the antenna under test (AUT). The feed-probe considerably affects not only the resonant frequency but also the radiation pattern of the AUT^8–10^. Moreover, for on-chip antenna measurements, it is necessary to detach the AUT from the chip to supply the RF signal for the measurements; therefore, the impact^11^ of the coupling between the AUT and the surrounding peripheral circuits cannot be evaluated accurately.

In our measurement setup (Fig. 1(b)), the spatial distributions of the amplitude and phase of the far-field, as well those of the near-field, are measured using two electro-optic (EO) probes: the measurement probe is for mapping and the reference probe for phase noise cancellation. Because we do not use metallic components such as feeding cables and RF probes but use metal-free EO crystals attached to the optical fiber, the field invasiveness is drastically minimized compared to the conventional technique^12^. Moreover, the frequency and phase fluctuations of the RF signal are precisely cancelled-out by the electronics. Thus, the standard deviations of the phase measurements attain the theoretical values, limited by the signal-to-noise ratio (SNR) of the amplitude measurements.

## Measurement Principle

**Figure 2:**
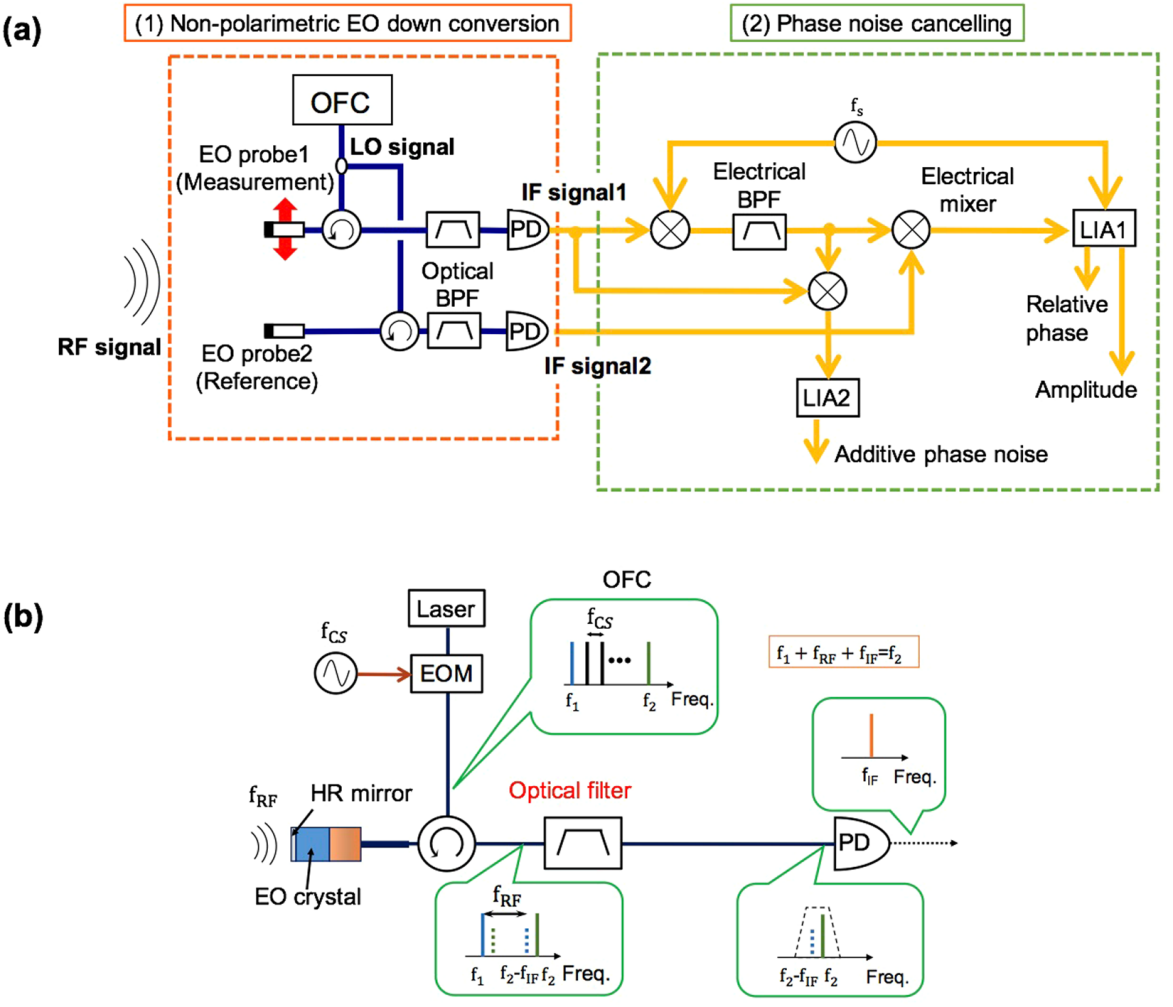
Basic configuration of the measurement setup. (**a**) System block diagram and (**b**) principle of the non-polarimetric EO frequency down conversion.

Figure 2 shows the basic configuration of the measurement setup. Our system consists of two parts: the non-polarimetric EO frequency down conversion part^13^ and the phase noise cancellation part. The non-polarimetric EO frequency down conversion part consists of 1.55 *μ*m telecom components. EO probe1 can be moved for mapping, whereas, EO probe2 is fixed at the reference point for phase reference measurement.

For the EO frequency down conversion, an optical frequency comb (OFC) is used for the LO signal, as shown in Fig. 2(b). Each comb mode is phase modulated by the RF signal to be measured, in the EO crystal and the sidebands are generated. The frequency down conversion principle is based on the coherent detection of the generated sideband^14^. One of the sidebands and the adjacent comb-mode pair are extracted by an optical filter and detected by a low-speed photodiode. The frequency of the intermediate frequency (IF) signal is, ${{\rm{f}}}_{{\rm{IF}}}={{\rm{f}}}_{2}-{{\rm{f}}}_{1}-{{\rm{f}}}_{{\rm{RF}}}={{\rm{mf}}}_{{\rm{CS}}}-{{\rm{f}}}_{{\rm{RF}}}$, where m is the mode separation index, f_cs_ is the comb-mode separation, and f_RF_ is the frequency of the RF signal. The frequency of the IF signal fluctuates, reflecting the relative frequency fluctuation between the RF signal, f_RF_ and the LO signal, mf_CS_. On the other hand, the frequency converted IF signal1 and signal2 have the same frequency fluctuations. This common mode frequency fluctuation, more generally the common mode phase noise, is cancelled-out in the second part of the system to extract the relative phase difference of the field measured at two different points^15^.

Let ${A}_{1}\,\cos ({\omega }_{IF}(t+{\rm{\Delta }}{\rm{\tau }})+{\rm{\Delta }}\varphi +{\varphi }_{n}(t+{\rm{\Delta }}{\rm{\tau }}))$ and ${A}_{2}\,\cos ({\omega }_{IF}t+{\varphi }_{n}(t))$ be IF signal1 and IF signal2, respectively, where Δτ and Δ*ϕ* are the temporal difference and the relative phase between the RF signal measured by EO probe1 and EO probe2, respectively; *ϕ*_*n*_ is the relative phase fluctuation between the RF and LO signals. For simplicity, we assume that there is no time delay, Δτ = 0. In the phase noise cancellation stage, the frequency of IF signal1 is up-converted by f_s_, using the first electrical mixer. With the electrical bandpass filter (BPF), we extracted the sum components, which are proportional to ${A}_{1}\,\cos (({\omega }_{IF}+{\omega }_{s})t+{\rm{\Delta }}\varphi +{\varphi }_{n}(t)+{\varphi }_{na}(t))$, where *ϕ*_*na*_(*t*) is the additive phase noise imposed by the BPF owing to the frequency fluctuations of IF signal1. This up-converted signal is then mixed with IF signal2 to generate a signal proportional to ${A}_{1}{A}_{2}\,\cos ({\omega }_{s}t+{\rm{\Delta }}\varphi +{\varphi }_{na}(t))$. With this double-stage mixing process, the common mode phase noise, *ϕ*_*n*_(*t*), is cancelled-out and only the phase difference between IF signal1 and signal2, Δ*ϕ* and additive phase noise, *ϕ*_*na*_(*t*), added in the system, are measured by lock-in detection with a local reference signal, f_s_. The additive phase noise is measured by lock-in amplifier2 (LIA2) and subtracted from the relative phase measured by LIA1.

## Results and Discussion

We demonstrate the capability of our system by measuring the mm-wave generated by a free-running self-oscillating Gunn oscillator. The frequency of the Gunn oscillator is approximately 77 GHz with a ±300 kHz short-term frequency fluctuation and a long-term frequency drift of several MHz.

**Figure 3:**
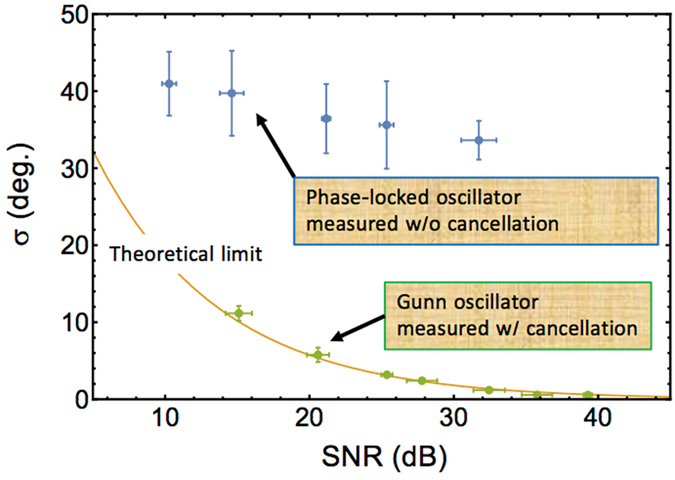
Relationship between the phase standard deviation and the amplitude SNR.

Figure 3 shows the relationship between the standard deviation of the relative phase measurement, σ and the SNR of the amplitude measurement. Amplitude noise, σ_*A*_, can be converted to phase noise, σ, with a maximum conversion ratio of ${\rm{A}}={\sigma }_{{\rm{A}}}/\sigma$, where A is the amplitude of the signal. Therefore, the theoretical curve is calculated using ${\rm{\sigma }}={10}^{-{\rm{SNR}}\mathrm{/20}}$ (rad) in the high SNR region, where ${\rm{SNR}}=20\,{\mathrm{log}}_{10}({\rm{A}}/{\sigma }_{{\rm{A}}})$. Owing to precise phase noise cancellation, the relative phase measured by our system agrees well with the theoretical curve. For comparison, we also plot the phase-measurement standard deviation of the mm-wave (77 GHz) generated by the eight-time multiplexing of a stable microwave signal from a signal source (Keysight: N5183B). The signal source and the synthesizer (Rohde & Schwarz: SMF100A) used for the OFC generation are mutually phase-locked via a 10 MHz reference. This measurement setup emulates the conventional measurement setup shown in Fig. 1(a), except for the EO probe used for the measurements. The residual phase noise between the synthesizers is multiplied as per the frequency multiplications; therefore, the standard deviation of the phase measurement does not attain the theoretical limit.

**Figure 4:**
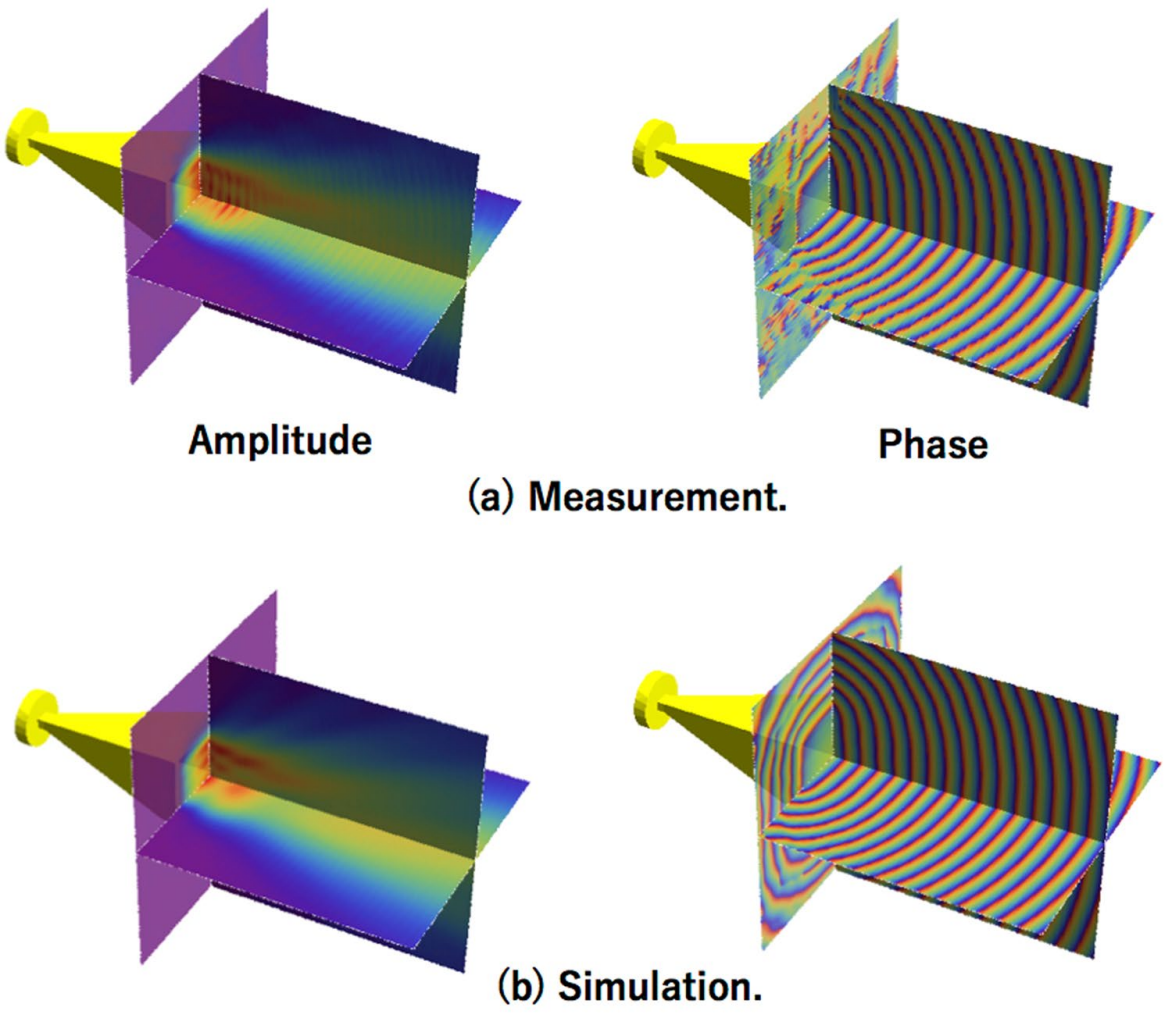
Amplitude and phase distribution of the mm-wave emitted by a horn antenna. The signal source is a free-running Gunn oscillator. (**a**) Measured results and (**b**) simulated results. Note: The pyramidal horn antennas are simulation models.

Figure 4(a) shows the mapped amplitude and relative phase distributions of the mm-wave generated by the Gunn oscillator. In this proof-of-concept experiment, the mm-wave was divided by a rectangular-waveguide-type power divider. Port1 of the divider was connected to the pyramidal horn antenna, whereas, port2 was kept open for the phase reference measurement. Note that the pyramidal horn antennas in Fig. 4 are simulation models, i.e., Fig. 4(a) is produced by merging the simulation model and the experimental results. As depicted here, the measured amplitude and phase distribution agree well with the simulated distribution.

**Figure 5:**
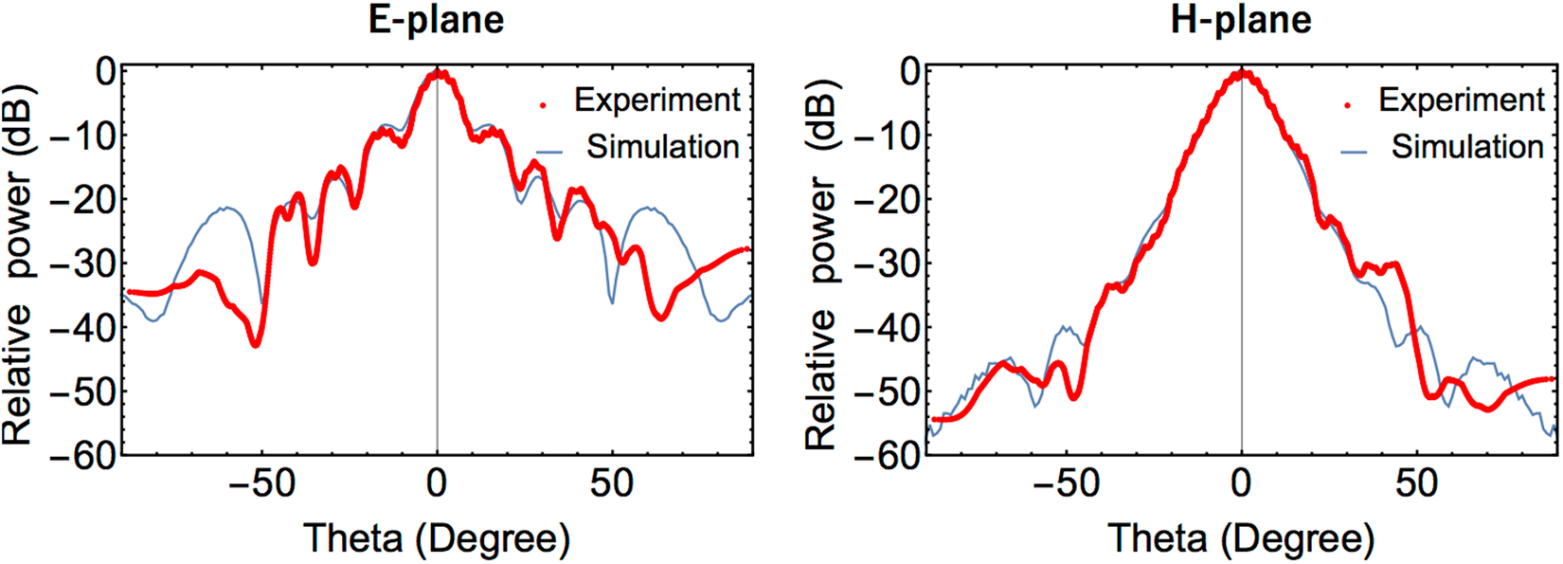
Radiation patterns of the horn antenna calculated from the measured near-field.

Figure 5 displays the far-field radiation pattern calculated from the measured near-field pattern. The red lines present the experimental results, whereas, the blue lines indicate the simulation results. We compared the measured antenna characteristics with those obtained by simulation. Tables 1 and 2 summarize the antenna characteristics in the E- and H-planes, respectively. The standard error of the measurements was calculated based on five independent measurements and the Student’s t distribution was 2.776, corresponding to a confidence interval of 95%. Owing to precise phase noise cancellation and minimum measurement disturbances, the experimentally obtained characteristics agree well with the results obtained by simulation. This indicates that our asynchronous system can be used to characterize the radiation patterns of on-chip antenna devices without antenna detachment.

**Table 1:** Radiation pattern characteristics in the E-plane.

	3dB-beamwidth	+1st sidelobe position	−1st sidelobe position
Simulation	10.3 (deg.)	14.9 (deg.)	−14.9 (deg.)
Experiment	9.6 ± 0.7 (deg.)	14.6 ± 1.7 (deg.)	−14.5 ± 0.3 (deg.)

**Table 2:** Radiation pattern characteristics in the H-plane.

	3dB-beamwidth	+1st sidelobe position	−1st sidelobe position
Simulation	11.9 (deg.)	35.7 (deg.)	−35.7 (deg.)
Experiment	10.8 ± 0.9 (deg.)	35.0 ± 2.9 (deg.)	−38.7 ± 3.0 (deg.)

## Conclusion

We have proposed and demonstrated a new scheme for mapping the spatial distribution of the amplitude and phase of an electromagnetic wave generated by a free-running self-oscillating generator. Using a wide-band OFC, our system can be applied to the 300-GHz band or higher, wherein, on-chip antenna devices for THz wireless communication have been extensively studied^16^. Our measurement concept can be applied to electromagnetic waves generated by any type of process, including nonlinear processes such as free-space THz comb radiation^17^.

## Methods

A 4-N,N-dimethylamino-4′-N′-methyl-stilbazolium tosylate (DAST) EO crystal was used. The DAST crystal (1 mm × 1 mm × 1 mm) and GRIN lens were mounted on a polarization-maintaining fiber (PMF). The polarization direction of the probe beam (slow-axis of the PMF) and the a-axis of the DAST crystal were in parallel. The diameter of the collimated probe beam in the EO crystal was 0.2 mm, limiting the ultimate spatial resolution.

The OFC, as the LO signal, was generated by the phase modulation of a 1.55 *μ*m continuous wave (CW) laser light. The comb-mode separation and the mode separation index were 12.93 GHz and m = 6, respectively. A THz-wide OFC can be generated by deep phase and/or amplitude modulation^18^. A mode-locked laser can also be used as an OFC with a bandwidth of several THz. However, the measurement bandwidth is limited by the comb-mode separation, f_CS_ because all the frequencies, mf_CS_ + f_RF_, emitted by the device should result in the same IF signal and cannot be distinguished.

The IF frequency was approximately 6.6 MHz. IF signal1 was mixed with a stable local reference signal, f_s_ = 4.7 MHz. The up-converted IF signal1 was extracted using a BPF with a 5-MHz bandwidth from 8.6–13.6 MHz and then mixed with IF signal2 to cancel-out the common-mode phase noise. However, the frequency fluctuations of the IF signals cause additive phase noises because of the frequency-dependent phase shift added by the BPF. This additive phase noise cannot be cancelled-out by the double-stage mixing process. In our system, this additive phase noise was extracted by mixing the up-converted IF signal1 with IF signal1 itself and detected using LIA2. The additive phase noise measured by LIA2 was subtracted from the phase measured by LIA1 to obtain the precise relative phase of the EM field.

Currently, the noise-cancellation bandwidth in the proof-of-concept system is limited to approximately 5 MHz. This bandwidth can be extended by changing the IF frequency and using a wider-bandwidth analog bandpass filter. Ultimately, the canceling bandwidth will be limited by the finite temporal difference, Δτ, between the RF signals measured by probe1 and probe2. If we apply the current setup to measure the far-field (~2D^2^/*λ*, where D = 33.8 mm is the maximum dimension of the horn antenna, and *λ* = 3.86 mm) without changing the reference point, the maximum canceling bandwidth will be limited to approximately 400 MHz.

The SNR of the measurements can be improved by minimizing the conversion losses of the mixers. Digital signal processing can be a promising option for noise cancellation to improve the SNR and bandwidth, simultaneously.

## Electronic supplementary material

Supplementary information
