# Supplementary material for: Mapping of electromagnetic waves generated by free-running self-oscillating devices
Source: Sci Rep. 2017 Aug 23;7:9203. doi: 10.1038/s41598-017-09802-0 (PMC5569046; doi:10.1038/s41598-017-09802-0)
Supplement: Supplementary file 1 — Supplementary information [file 41598_2017_9802_MOESM1_ESM.pdf]

SUPPLEMENTARY INFORMATION

Mapping of electromagnetic waves generated by free-running self-oscillating devices

Shintaro Hisatake[1], Hikaru Nakajima[2], Hai Huy Nguyen Pham[2], Hirohisa Uchida[3], Makoto Tojyo[4], Yoichi Oikawa[4], Kunio Miyaji[4], and Tadao Nagatsuma[2]

[1]Gifu University, Department of Electrical, Electronic and Computer Engineering, Gifu, 501-1193, Japan
[2]Osaka University, Department of Systems Innovation, Osaka, 560-8531, Japan
[3]Arkray Inc., Kyoto, 602-0008, Japan
[4]Think-Lands Co., Ltd., Yokohama, 230-0046, Japan

1 Frequency fluctuation of the Gunn oscillator used in the experiments

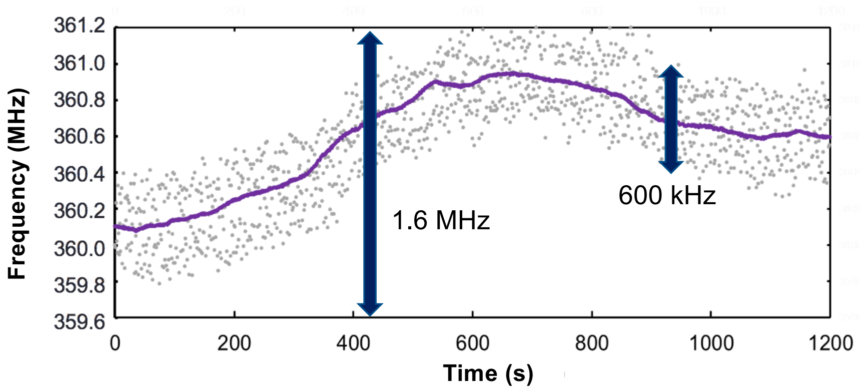

Figure A. Frequency fluctuation of the mm-wave generated by the Gunn oscillator.

Figure A shows the frequency fluctuations of the mm-wave generated by the Gunn oscillator. The mm-wave was down-converted by a mixer to an IF frequency of 360 MHz. The frequency of the IF signal was measured using a microwave counter with a 1-s interval. The solid line in Fig. A represents the smoothened data with a 30-s moving average. The frequency deviation was within the noise cancellation bandwidth.

2 Near-to-far field transformation

The measured amplitude and phase information on the XY-plane at z_0=10 mm from the horn antenna surface, depicted in Fig. B, were used for the near-to-far field transformation process. The maximum dimension of the horn antenna was D=33.8 mm and the mm-wave wavelength was λ=3.86 mm; therefore, the measured XY-plane was located in the radiating near-field region, where $\lambda/2 < z_0 < 2D^2/\lambda$. The measured area was 50 $\times$ 50 mm and the sampling interval was 0.2 mm ($\approx 0.05\,\lambda$), which is smaller than $\lambda/2 = 1.95$ mm; therefore, it satisfies the Nyquist criterion. The spatial frequency spectrum can be calculated from the Fourier transformation of the near-field electric-field distribution, as per the following equation:

$$\mathrm{F}(\mathrm{k_x},\mathrm{k_y}) = \frac{1}{2\pi}\int_{-\infty}^{\infty}\int_{-\infty}^{\infty}\mathrm{E}(\mathrm{x},\mathrm{y},\mathrm{z_0})\mathrm{e}^{-\mathrm{j}\mathrm{k_x}\mathrm{x}}\mathrm{e}^{-\mathrm{j}\mathrm{k_y}\mathrm{y}}\mathrm{dxdy}, \tag{1}$$

where $\mathrm{E}(\mathrm{x},\mathrm{y},\mathrm{z_0})$ is the distribution of the electric-field over a scanning surface at $\mathrm{z} = \mathrm{z_0}$; $\mathrm{k_x} = \mathrm{k}\ \sin\theta\ \cos\phi$ and $\mathrm{k_y} = \mathrm{k}\ \sin\theta\ \sin\phi$ are the wavenumber components in the X- and Y-directions, respectively. θ and ϕ are the angles in spherical coordinate representation. $\mathrm{k} = 2\pi/\lambda$ is the wavenumber.

Fig. C shows the three-dimensional (3D) radiation patterns of the pyramidal horn antenna, obtained by the transformation of the measured near-field patterns shown in Fig. B. The E- and H-planes in Fig. **??** were obtained by cutting the cross sections of Fig. C in each plane, respectively. We used the well-known Whittaker-Shannon interpolation method, based on the summation of the sinc function at each data point. Note that the sampling interval of the near-field measurement satisfies the Nyquist criterion; hence, the interpolation method can be applied.

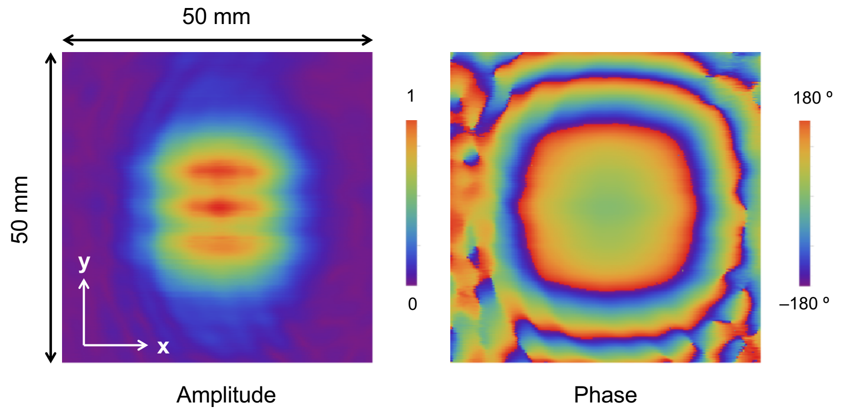

Figure B. Measured amplitude and phase distribution of the mm-wave in the XY-plane.

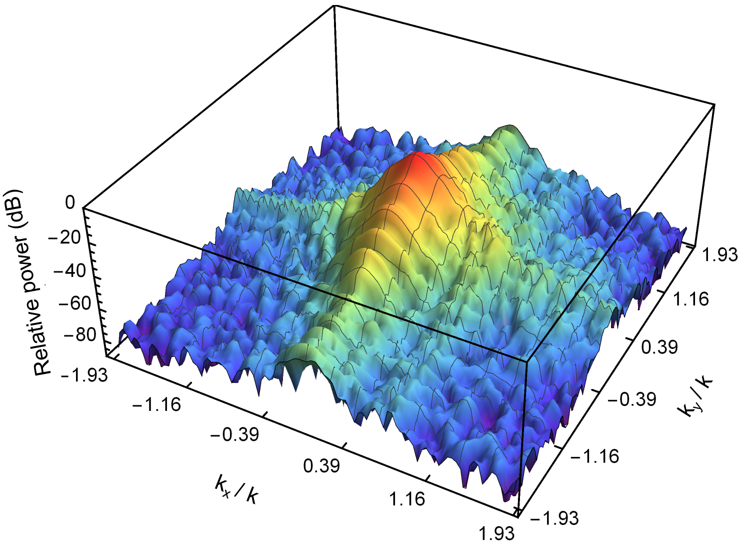

Figure C. Three-dimensional radiation patterns of the pyramidal horn antenna obtained by the transformation of the measured near-field patterns.
